# Insights Into the Role of the Lung Virome During Respiratory Viral Infections

**DOI:** 10.3389/fimmu.2022.885341

**Published:** 2022-04-27

**Authors:** Bárbara N. Porto

**Affiliations:** ^1^ Department of Medical Microbiology and Infectious Diseases, Rady Faculty of Health Sciences, University of Manitoba, Winnipeg, MB, Canada; ^2^ Biology of Breathing Group, Children’s Hospital Research Institute of Manitoba, Winnipeg, MB, Canada

**Keywords:** lung virome, microbiome, respiratory virus, virus infection, disease pathogenesis

## Abstract

The virome constitutes the viral component of the microbiome and it consists of the genomes of all the viruses that inhabit a particular region of the human body, including those that cause acute, persistent or latent infection, and retroviral elements integrated to host chromosomes. The human virome is composed by eukaryotic viruses, bacteriophages and archaeal viruses. The understanding of the virome composition and role on human health has been delayed by the absence of specific tools and techniques to accurately characterize viruses. However, more recently, advanced methods for viral diagnostics, such as deep sequencing and metagenomics, have allowed a better understanding of the diverse viral species present in the human body. Previous studies have shown that the respiratory virome modulates the host immunity and that, since childhood, the human lung is populated by viruses for whom there is no disease association. Whether these viruses are potentially pathogenic and the reason for their persistence remain elusive. Increased respiratory viral load can cause exacerbation of chronic pulmonary diseases, including COPD, cystic fibrosis, and asthma. Moreover, the presence of resident viral populations may contribute to the pathogenesis of community-acquired respiratory virus infections. In this mini review, I will discuss the recent progress on our understanding of the human lung virome and summarize the up-to-date knowledge on the relationships among community-acquired respiratory viruses, the lung virome and the immune response to better understand disease pathophysiology and the factors that may lead to viral persistence.

## Introduction

**Figure 1:**
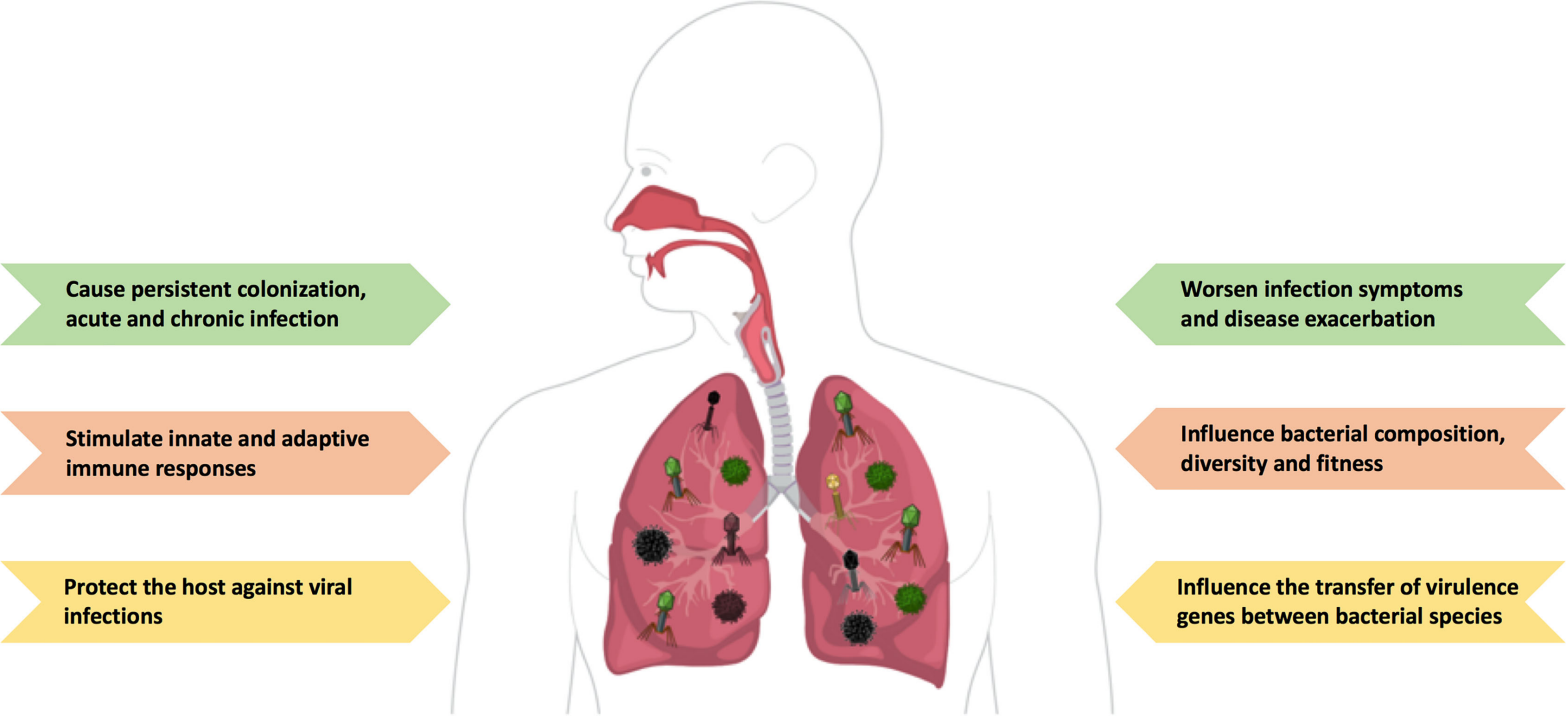
The ambiguous role of the lung virome during pulmonary viral infections. The human lung is populated by different viruses and their genomes (called ‘the virome’), including DNA and RNA viruses, and bacteriophages. The lung virome has been shown to have a protective role by stimulating a continuous antiviral response. However, higher viral loads may have harmful consequences to the host and exacerbate disease pathogenesis, either infectious or non-infectious. Figure created with https://mindthegraph.com/.

The virome constitutes the viral component of the human microbiome and it consists of the genomes of all the viruses that inhabit a particular region of the human body, including those that cause acute, persistent, or latent infection, and retroviral elements integrated to host chromosomes. The human virome is composed by eukaryotic viruses (viruses that infect eukaryotic cells), bacteriophages (viruses that infect human-hosted bacteria) and archaeal viruses (viruses that infect archaea) (1–3). Although significant progress has been made in sequencing technology, studies addressing the non-bacterial components of the human microbiome are still scarce, including the virome. Viruses lack conserved regions such as bacterial 16S or fungal 18S genes; therefore, most studies addressing the virome use targeted detection of specific viruses, whereas a more comprehensive characterization of the virome requires shotgun sequencing (4). A meta-analysis study revealed that the human virome is represented by 320 viral species classified in 26 families. Of these, *Anelloviridae*, *Papillomaviridae*, and *Bunyaviridae* are the most abundant and account for 44% of all human viruses (5). Recent studies suggest that the human virome comprehends commensals and opportunistic pathogens. The balance between being a commensal or becoming a pathogen is determined by different factors of the viral community itself and the host, such as genetic factors and the immune status (1). Previous studies have shown that the respiratory virome modulates the host immunity and that, since childhood, the human lung is populated by viruses for whom there is no disease association. However, whether these viruses are potentially pathogenic and the reason for their persistence remain elusive (**Figure 1**). There is evidence showing that the lung viral inhabitants play a critical role in modulating and priming the host immune response. The presence of different transitory viruses continuously stimulates an antiviral immunity, which may confer an advantage to the host by protecting against potentially pathogenic viral infections (2, 6). However, an increase in respiratory virus load or a suppression in the host antiviral responses may affect host physiology and cause exacerbations of chronic pulmonary diseases such as asthma, COPD, and cystic fibrosis (7, 8), which can contribute to the pathogenesis of these conditions. Here, I discuss the recent progress on our understanding of the human lung virome and summarize the up-to-date knowledge on the relationships among community-acquired respiratory viruses, the lung virome and the immune response to better understand disease pathophysiology and the factors that may lead to viral persistence.

## Bacteriophages and Their Role During Pulmonary Viral Infections

Bacteriophages (or phages) are obligate parasites of bacteria and develop complex interactions with their hosts. Therefore, their survival and fitness depend on their bacterial hosts (9). Several microbiota studies have demonstrated that bacteriophages are associated with mucosal tissues of healthy subjects (10–12). It has been suggested that bacteriophages play a critical role on the composition and burden of the bacterial microbiota, promoting dysbiosis and likely shaping the host’s immune system. For example, bacteriophages serve as reservoirs of genetic diversity within the bacterial microbiota as they influence the transfer of virulence genes between bacterial species. Most bacterial virulence factors, including bacterial colonization, adhesion, invasion, and even toxins, are encoded by bacteriophages (13, 14). Hence, it is plausible to assume that bacterial acquisition of such genes may alter microbiota diversity and functionality and influence host immunity. Besides having an indirect effect on host immunity through their relationships with bacteria, phages can also directly interact with host cells. It has been previously reported that dendritic cells actively phagocytose phage particles (15). Dendritic cell phagocytosis of *Pseudomonas aeruginosa* phages has been shown to trigger Toll-like receptor 3 (TLR-3)- and TIR domain-containing adapter-inducing interferon β (TRIF)-dependent type I interferon production, promoting an antiviral response (16). Phages are also able to induce a pro-inflammatory response by activating macrophages and inducing the release of IL-1β and TNF (17). Moreover, phages inhibit the activation and proliferation of human T cells *\in vitro* (18). Phages and their nucleic acids are able to change the expression of innate immune genes in different mouse tissues (17, 19). Recently, Nguyen et al. have demonstrated the directional transcytosis of different phages across epithelial cell layers. This study revealed that epithelial cells took up and transported phages through both the vesicular and cytosolic compartments and released active phages on the opposite cell surface (20). The underlying mechanisms of phage transcytosis have yet to be demonstrated, but probably include pattern recognition of phage structural proteins by epithelial cells. Further studies are needed to elucidate the mechanisms of phage–eukaryotic cell interactions, however these studies suggest that phages physically interact and stimulate the host immune response.

Although it is well described that there is a high prevalence of phages in the respiratory tract (8, 21, 22), the literature regarding the composition and roles of bacteriophages during respiratory viral infections is limited. In 2011, one of the first studies to characterize the respiratory virome in nasopharyngeal swabs from children with acute lower respiratory tract infections detected over 100 different species of virus; of these, the majority - 89 - corresponded to bacteriophage sequences and were excluded from further analyses (23). In contrast to the high abundance of bacteriophages found in the 2011 study, Lyshom and colleagues, using viral metagenomic sequencing of respiratory tract secretions from hospitalized patients with severe lower respiratory tract infections observed that only a small portion of the samples’ content was comprised by bacteriophages (24). More recently, Li and colleagues compared the respiratory virome and serum cytokine profile in a population of 4407 children diagnosed with acute respiratory tract infections (ARTIs) over a 6-year period. The relative abundance of *Propionibacterium* phages was significantly elevated in children with multiple ARTIs compared to those children with a single ARTI. Serum levels of tissue inhibitor of metalloproteinases-1 (TIMP-1) and platelet-derived growth factor subunits BB (PDGF-BB) were increased in children with multiple ARTIs compared to those with a single ARTI and non-ARTI controls. Furthermore, the presence of *Propionibacterium* phages correlated with higher levels of these cytokines (25). These data suggest that an increased abundance of *Propionibacterium* phages and higher levels of TIMP-1 and PDGF-BB may be associated with the onset of multiple episodes of ARTI in children over time. The lack of studies addressing the role of bacteriophages on lung dysbiosis and their direct interaction with the host immune system during pulmonary viral infections has been neglected and warrants further investigations.

## Eukaryotic Viruses and Their Pathologic Association With Respiratory Viral Infections

Although most respiratory viruses and their pathogenic effects have been well studied, the implications of the viruses detected in different regions of the respiratory tract and the reasons for their persistence are not completely understood. Several studies have demonstrated that the airways of healthy children and adults are populated with DNA and RNA viruses, and bacteriophages (8, 26–28). Moreover, the recently established family of DNA viruses, called *Anelloviridae*, represents almost 70% of all viruses detected in blood and in most tissues of healthy subjects (29). So far, anelloviruses have not been associated with disease and whether these viruses have a pathogenic role in the human lungs remains elusive. However, it has been suggested that increased anellovirus load is associated with immunosuppression (30).

Adult patients with severe lower respiratory tract infections (LRTIs) have been shown to exhibit an increased virus diversity in their airways. A study has analyzed nasopharyngeal aspirates of 210 patients with LRTIs and identified 39 virus species, with *Paramyxoviridae*, *Orthomyxoviridae*, and *Picornaviridae* being the most common virus families observed. Within these families, the most prevalent viruses were respiratory syncytial virus (RSV), metapneumovirus, rhinovirus-A (RV-A), RV-B, RV-C, influenza A and influenza B (24). Together with these common viruses, less well represented viruses were also observed, including bocavirus, polyomavirus and torque-teno vius (TTV) (24). A prospective multicentre study that enrolled 2000 adult pneumonia patients has detected rhinoviruses and influenza viruses in 9% and 6% of the patients’ samples, respectively (31). The same group investigated the presence of respiratory viruses in samples from more than 2000 children with community-acquired pneumonia and found that RSV, rhinoviruses, metapneumovirus, and adenovirus were the most common pathogens (32). These studies identified key pathogenic viruses present in airway samples of adults and children with community-acquired pneumonia and the viruses detected are likely to play a role on disease pathogenesis associated with pneumonia. Noteworthily, hospitalized patients undergoing mechanical ventilation, regardless of acute respiratory viral infection, have been reported to present several viruses in their bronchoalveolar lavage (BAL) samples. Viruses such as RV, RSV, influenza virus, parainfluenza virus, and metapneumovirus were observed in 30% of BAL samples of patients negative for respiratory infection, while influenza virus, parainfluenza virus, RV, RSV, metapneumovirus, bocavirus, and enterovirus were detected in 50% of the samples from patients diagnosed with respiratory infections (33). Recently, a new family of small circular DNA viruses, called *Redondoviridae*, was identified and characterized in oropharyngeal samples from patients with periodontal disease and in endotracheal aspirates from critically ill patients in intensive care units. Although redondovirus was also found in samples of healthy individuals, critically ill patients presented significantly higher amounts of the virus genome in their aspirates. Importantly, redondovirus was detectable over a period of 2–3 weeks, suggesting persistent colonization or infection (34). Moreover, redondovirus was detected in 12% of nasopharyngeal samples, 8% of sputum samples, and 4% of pharyngeal swabs from patients hospitalized for respiratory viral infections of different etiologies. Interestingly, the virus could not be detected in blood samples of those patients, suggesting that redondovirus may be restricted to the respiratory tract (35).

To investigate the role of the respiratory virome in children with unexplained fever, two studies published on the same year enrolled both febrile and afebrile young children and tested their nasopharyngeal swabs for common respiratory pathogens, including influenza A virus, parainfluenza virus, metapneumovirus, rhinovirus, coronavirus, enterovirus, adenovirus, bocavirus, and the recently discovered KI and WU polyomaviruses. Overall, the studies have found the predominance of pathogens such as adenovirus, enterovirus, human herpesvirus 6, roseoloviruses, and parechovirus in the nasopharyngeal samples from febrile children compared to the ones from afebrile children. Also, samples from febrile children contained a broader range of viral genera and exhibited multiple viral genera more frequently than samples from afebrile children (26, 36). These studies suggest that virus infection is associated with unexplained fever in young children and may inform rational medical treatment of febrile children by limiting the unnecessary use of antibiotics. Additionally, viral diversity has been shown to be greater in nasopharyngeal samples from children hospitalized for severe acute respiratory infections (SARIs). Whereas nasopharyngeal samples from children with SARI are composed by viruses from the *Paramyxoviridae*, *Coronaviridae*, *Parvoviridae*, *Adenoviridae*, *Orthomyxoviridae*, *Picornaviridae*, and *Anelloviridae*, those from healthy children are composed by anelloviruses and bacteriophages (37). This study provides evidence for the differences in viral composition and diversity during acute infection compared to healthy state. To date, studies that have attempted to determine the composition of the human respiratory virome indicate that a common virome signature is present in the respiratory tract in both health and disease states. Nevertheless, during infectious disease, the virome burden increases and may be associated to the development of a severe condition.

## SARS-CoV-2 and the Virome

From December 2019, severe acute respiratory syndrome coronavirus 2 (SARS-CoV-2) infection became a pandemic, affecting more than 240 million people worldwide and causing over 4.9 million deaths, and unprecedented social and economic disruption (38, 39). Most of the infected individuals exhibit mild upper respiratory tract symptoms, such as fever, fatigue, and dry cough. However, some people, including the elderly and those with chronic diseases, can develop severe lower respiratory tract symptoms (40), which may progress to respiratory failure due to the exaggerated acute lung injury (41). Some coronavirus disease-19 (COVID-19) patients have been reported to present a cytokine storm syndrome, which seems to be involved in multi-organ failure (42).

Understanding COVID-19 pathogenesis has been a research priority worldwide. Whereas the literature regarding the bacterial microbiome in COVID-19 patients has significantly evolved, studies reporting the composition of both the respiratory and gut virome are still scarce. A recent study with 19 COVID-19 patients revealed that the gut virome of these patients is enriched with *Herelleviridae*, *Virgaviridae*, and bacteriophages of several different families compared with healthy individuals, suggesting gut dysbiosis (43). Regarding the lung virome, Merenstein and colleagues examined oropharyngeal samples from 83 hospitalized COVID-19 patients as well as non-COVID-19 patients and healthy controls for the presence of bacterial communities and the DNA virus families *Anelloviridae* and *Redondoviridae*. The authors found an abundance of both *Anelloviridae* and *Redondoviridae* in patients’ samples and the presence of these viruses positively correlated with intubation during hospitalization (44). A more recent study analyzed nasopharyngeal swabs from 89 patients in Italy during the three different COVID-19 waves (March-May, 2020; September-November, 2020; January-February, 2021). The authors detected 6 virus families in the samples, including *Retroviridae*, *Herpesviridae*, *Poxviridae*, *Pneumoviridae*, *Pandoraviridae*, and *Anelloviridae*. However, the predominance of these viruses was not associated with disease severity (45). The investigation of the respiratory virome during COVID-19 is still in its infancy; nonetheless, these recent studies shed some light on virus composition and likely co-infection, and may open new avenues for future studies to address clinical outcomes of airway dysbiosis in SARS-CoV-2 infection and the role of the lung virome on COVID-19 pathophysiology.

## Conclusion

**Figure 2:**
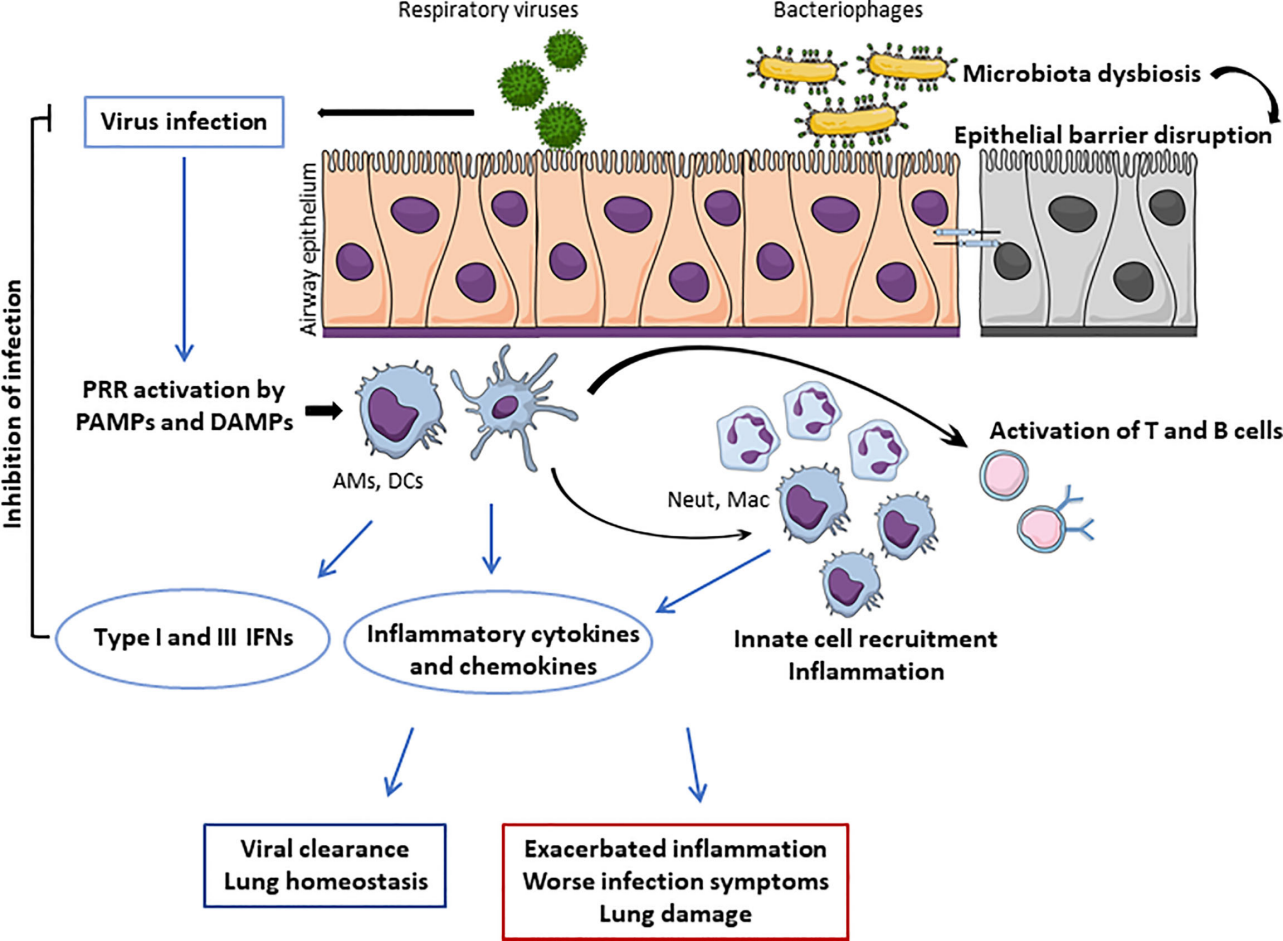
Overview of beneficial and harmful roles of the lung virome during respiratory viral infections. Respiratory viruses are recognized by pattern-recognition receptors (PRR) expressed by both the airway epithelial cells and resident immune cells, such as alveolar macrophages (AMs) and dendritic cells (DCs). Once activated, AMs and DCs secrete pro-inflammatory cytokines and chemokines, which promote a local inflammatory response and the recruitment and activation of leukocytes, including neutrophils (Neut), and macrophages (Mac). Furthermore, AMs and DCs secrete type I and III interferons (IFNs), leading to virus clearance. Also, antigen-presenting cells, such as DCs, activate the adaptive immunity, mediated by T and B cells. This tightly controlled response leads to viral clearance and return to lung homeostasis. However, high lung viral loads along with microbiota dysbiosis and disruption of epithelial barrier can lead to an exacerbated inflammatory response, worsen infection symptoms, and ultimately lung damage. Figure created with MindtheGraph.com.

Although significant progress has been made in the respiratory microbiome research field, there is still a limited number of studies addressing the composition, diversity, and particularly the role of the lung virome on respiratory tract infections. It is becoming increasingly apparent that the human respiratory virome influences health and disease as much as the bacterial mucosal inhabitants. Moreover, the intricate relationship of bacterial viruses and their hosts impacts the human body by affecting bacterial diversity, and consequently causing dysbiosis, likely promoting further harm to the infected human host (**Figure 2**). Associations between the changes in the lung virome and severity of disease, either infectious or non-infectious, have been increasingly common; however, studies addressing direct causality and the underlying mechanisms for such associations are still lacking. Future investigations focused on the lung virome will provide us with a better understanding of the viral populations living within our respiratory tract and their interplay with the bacterial microbiome and the host’s immune system.

## Author Contributions

BP: study design, literature search, data collection, and manuscript writing.

## Funding

This study was supported by University of Manitoba Start-Up Funds to BNP.

## Conflict of Interest

The author declares that the research was conducted in the absence of any commercial or financial relationships that could be construed as a potential conflict of interest.

## Publisher’s Note

All claims expressed in this article are solely those of the authors and do not necessarily represent those of their affiliated organizations, or those of the publisher, the editors and the reviewers. Any product that may be evaluated in this article, or claim that may be made by its manufacturer, is not guaranteed or endorsed by the publisher.
